# Bovine Teat Microbiome Analysis Revealed Reduced Alpha Diversity and Significant Changes in Taxonomic Profiles in Quarters with a History of Mastitis

**DOI:** 10.3389/fmicb.2016.00480

**Published:** 2016-04-08

**Authors:** Hélène Falentin, Lucie Rault, Aurélie Nicolas, Damien S. Bouchard, Jacques Lassalas, Philippe Lamberton, Jean-Marc Aubry, Pierre-Guy Marnet, Yves Le Loir, Sergine Even

**Affiliations:** ^1^Institut National de la Recherche Agronomique, UMR 1253 STLORennes, France; ^2^Agrocampus Ouest, UMR 1253 STLORennes, France; ^3^Institut National de la Recherche Agronomique, UMR 1348 PEGASESaint-Gilles, France; ^4^Agrocampus Ouest, UMR 1348 PEGASERennes, France

**Keywords:** bovine microbiota, bovine microbiome, dysbiosis, mammary gland, dairy ruminant, mastitis

## Abstract

Mastitis is a mammary gland inflammatory disease often due to bacterial infections. Like many other infections, it used to be considered as a host-pathogen interaction driven by host and bacterial determinants. Until now, the involvement of the bovine mammary gland microbiota in the host-pathogen interaction has been poorly investigated, and mainly during the infectious episode. In this study, the bovine teat microbiome was investigated in 31 quarters corresponding to 27 animals, which were all free of inflammation at sampling time but which had different histories regarding mastitis: from no episode of mastitis on all the previous lactations (Healthy quarter, Hq) to one or several clinical mastitis events (Mastitic quarter, Mq). Several quarters whose status was unclear (possible history of subclinical mastitis) were classified as NDq. Total bacterial DNA was extracted from foremilk samples and swab samples of the teat canal. Taxonomic profiles were determined by pyrosequencing on 16s amplicons of the V3-4 region. Hq quarters showed a higher diversity compared to Mq ones (Shannon index: ~8 and 6, respectively). Clustering of the quarters based on their bacterial composition made it possible to separate Mq and Hq quarters into two separate clusters (C1 and C2, respectively). Discriminant analysis of taxonomic profiles between these clusters revealed several differences and allowed the identification of taxonomic markers in relation to mastitis history. C2 quarters were associated with a higher proportion of the Clostridia class (including genera such as *Ruminococcus, Oscillospira, Roseburia, Dorea*, etc.), the Bacteroidetes phylum (*Prevotella, Bacteroides, Paludibacter*, etc.), and the Bifidobacteriales order (*Bifidobacterium*), whereas C1 quarters showed a higher proportion of the Bacilli class (*Staphylococcus*) and Chlamydiia class. These results indicate that microbiota is altered in udders which have already developed mastitis, even far from the infectious episode. Microbiome alteration may have resulted from the infection itself and or the associated antibiotic treatment. Alternatively, differences in microbiome composition in udders with a history of mastitis may have occurred prior to the infection and even contributed to infection development. Further investigations on the dynamics of mammary gland microbiota will help to elucidate the contribution of this endogenous microbiota to the mammary gland health.

## Introduction

For decades now, the development of infections has been considered as the result of a bipartite interaction of a pathogen with a host. Development of high-throughput sequencing techniques has opened a new field of investigation that has made it possible to characterize microbiomes associated with hosts in greater depth and that has revealed a larger role than previously imagined for microbiota (Vayssier-Taussat et al., 2014). In animals, interest has increased in the exploration of microbiotas, notably for livestock species such as pigs, cattle and chickens, with regard to animal performance, genetics, diet and health (Sandri et al., 2014; Kim and Isaacson, 2015; Schokker et al., 2015; Weimer, 2015).

In cattle, major efforts have been devoted to the characterization of the rumen microbiome in the last few years in relation to diet, rumen development, fermentation efficiency including the production of greenhouse gases such as CO2 and methane, and animal performance (feed efficiency, milk composition; Jami et al., 2014, p. 2; McCann et al., 2014; Mohammed et al., 2014; Sandri et al., 2014; Henderson et al., 2015; Jewell et al., 2015; Kumar et al., 2015; Minuti et al., 2015; Myer et al., 2015; Veneman et al., 2015; Weimer, 2015). Two other microbiota have been explored, which are associated with the mammary gland and reproductive tract (uterus/vagina), in relation to the two main post-partum diseases in cattle, mastitis and metritis (Santos et al., 2011; Machado et al., 2012; Oikonomou et al., 2012, 2014; Santos and Bicalho, 2012; Knudsen et al., 2014; Rodrigues et al., 2015). Mastitis is an inflammation of the mammary gland that generally has an infectious origin. This inflammation is diagnosed through an increase of the somatic cell count (SCC) in milk, which mainly corresponds to neutrophil recruitment in the mammary gland. SCC increase can be associated with clinical signs including udder inflammation, abnormal milk or systemic signs such as fever (clinical mastitis) or the lack of clinical signs (subclinical mastitis). Mastitis is among the most common diseases in dairy cattle and affects animal welfare as well as productivity parameters (Nader-Macías et al., 2008). It has a huge impact on the economy of the dairy production chain (Heikkilä et al., 2012). Prevention and curative strategies that mainly rely on antibiotherapy are not fully effective at this time, frequently resulting in chronic and recurrent infections. This prompts the need of a better understanding of host (mammary gland)/pathogen interactions as a prerequisite for the development of efficient diagnostic tools and therapeutic interventions. In line with the concept of pathobiome (Vayssier-Taussat et al., 2014), this better understanding of the host pathogen interaction includes a better characterization of the mammary gland microbiome and of its role in disease development.

So far, the analysis of mammary gland microbiota was mainly undertaken to provide new insights into the ecology of species related to inflammatory disorders, by comparing the microbiomes of healthy cows with the microbiomes of cows undergoing inflammation (Oikonomou et al., 2012, 2014; Kuehn et al., 2013). Using high-throughput automated DNA pyrosequencing, these studies have explored the bacterial profile of milk isolated from quarters undergoing clinical and subclinical mastitis and from healthy quarters, giving new insights into the bacterial profiles associated with mastitis, notably for culture-negative samples. It can be observed that the above-mentioned studies shed light on milk microbiota composition at the time of infection. Apart from the infectious episode, the composition of the microbiota of quarters that develop mastitis has never been explored.

In the present study, we used high-throughput automated DNA pyrosequencing to explore the link between bovine teat microbiome composition and history of quarter with regard to mastitis. Sampling was performed on quarters that were free of mastitis at the time of sampling (no signs of inflammation and low SCC), but that had different histories with regard to mastitis. We focused on the microbiota located inside the teat (contained in foremilk and attached to the teat internal epithelium) since this microbiota constitutes the first potential microbiological barrier against pathogen entry.

## Materials and methods

### Experimental design

Sampling was performed at the INRA UMR PEGASE experimental farm on a Prim'Holstein herd. The cows were kept indoors in free stalls with an average per-cow surface area of 9 m2. They were fed twice daily at 08h00 and 15h00 with the same diet consisting of (percentage of dry mater in the diet): corn silage (64.7%), energy concentrate (14.8%), soybean meal (10.5%), dried alfalfa (10%). Cows were on twice-daily milking. Classical hygienic procedures included cleaning of teats with individual paper towel before milking and post milking teat dipping in iodine solution. The protocol was reviewed and approved by the Regional Ethics Committee for Animal Use and Care (Bretagne, France). Sampling is part of a classical veterinary practice. According to the European directive 2010/63/EU, this type of experiment does not require an authorization request.

Sampling was performed between December 2012 and March 2013, ~70 days after calving on cows with parity between two and four. Animals had not developed mastitis during ongoing lactation prior to sampling: no clinical signs of mastitis and SCC values (measured twice a week on the milk collected from the four quarters, i.e., composite milk) lower than 250,000 cells/ml during ongoing lactation. In addition, cows retained for the study had not received any local or systemic antibiotic treatment during the ongoing lactation, considering that recent antibiotic treatment could modify microbiota. However, they had all received dry cow antibiotic therapy at the end of the previous lactation. One quarter per cow was sampled to increase biodiversity, except for four cows for which two quarters were sampled. This was done to see whether taxonomic profiles from quarters corresponding to the same animal were closest than profiles from different animals. Only quarters with SCC values lower than 100,000 cells/ml 1 week before sampling and the day of sampling were retained. In total, 31 samples corresponding to 27 animals were retained. The designations of animals are indicated by V, followed by a number (e.g., V1, V2).

Quarters were classified based on the history of the animal and the quarter itself, taking all the lactations into account (Table S1). Briefly, quarters were classified as Healthy (Hq) when they had never encountered mastitis. These quarters correspond to animals that had no history of mastitis at all: no clinical signs of mastitis on the four quarters and SCC lower than 250,000 during all the lactations (measured twice a week on composite milk). Quarters were considered as Mastitis quarters (Mq) if they had already developed clinical mastitis (clinical signs associated to SCC increase and antibiotic treatment). All other quarters were classified as Not Determined status (NDq). These NDq quarters correspond to animals that had already undergone one or several increases of SCC (common in clinical and subclinical mastitis), but the sampled quarter has never developed clinical signs of mastitis (i.e., they are not Mq). In the case of subclinical mastitis, quarter(s) responsible for the increase of SCC is (are) generally not identified. In the case of clinical mastitis, we cannot exclude the possibility that quarters other than the Mq quarters developed subclinical mastitis concomitantly with the clinical mastitis.

### Sample collection

The sampling procedure was performed during the morning milking, essentially as previously described (Bouchard et al., 2015). Briefly, teats were thoroughly washed with osmosis water and cleaned with 70% ethanol and individual paper towels. Teat canals were then sampled in two different ways. Foremilk samples, corresponding to the milk stored in the teat cistern, were collected in sterile plastic tubes. A 5-mm sterile Histobrush® swab (D. Dutscher, Brumath, France), was then inserted for 5 mm inside the teat apex and turned three times before removal. The swabs were immediately placed in tubes containing foremilk and tubes were stored on ice until processing in the laboratory.

Approximately, 30 ml of the cisternal milk were further collected for SCC determination by LILLAB (Chateaugiron, France) and microbiological analysis: 100 μL were plated on Columbia II containing 5% sheep blood (BD, Le Pont de Claix, France) and aerobically incubated for 24–48 h at 37°C. Samples were considered as infected (I) in the presence of more than five colonies corresponding to the same morphology.

Foremilk samples were processed immediately on arrival at the laboratory. Following removal of the swab, the foremilk sample was mixed with 1/3 V of sodium citrate (1M, pH 7.5) and centrifuged (20 min, 4°C, 18,000 g). The pellet was washed in 1 ml of sodium citrate (20 g/L, pH 7.5), centrifuged (15 min, 4°C, 18,000 g), treated in 100 μL of 0.01% triton (Bouchard et al., 2013) and immediately resuspended in 900 μL PBS 1X and centrifuged (10 min, 4°C, 18,000 g). This last step made it possible to lyse bovine cells contained in foremilk samples and to remove released bovine DNA that could interfere with further steps due to its large quantity. The bacterial population was not altered by this step as checked during preliminary experiments by plating bacterial pellet on Plate Count Agar (Grosseron, Coueron, France) for 48 h at 30°C. The pellet was then stored at −20°C until DNA extraction.

### DNA extraction

Bacterial pellets were lysed for 45 min at 37°C in 360-μL lysis buffer containing 20 mM TRIS HCl (pH 8), 2 mM EDTA, 1% triton X100, 20 mg/mL Lysozyme (MP Biomedicals Illkirch, France), 50 U/mL mutanolysine (Sigma-Aldrich, Saint-Quentin Fallavier, France), and 200 μg/mL Lysostaphin (Sigma-Aldrich). Genomic DNA was purified using the DNeasy® Blood & Tissue Mini Kit (Qiagen, Courtaboeuf, France), according to the manufacturer's recommendations.

### PCR amplification of the V3-4 region of bacterial 16S rRNA genes and pyrosequencing of amplicons

PCR amplification of the V3-4 region of 16S rRNA genes was done using the universal primers S-D-Bact-0341-b-S-17 and S-D-Bact-0785-a-A-21. This primer pair gave an amplicon size of 464 bp and was identified as one of the best primer pairs for Bacteria and Archaea, giving the best overall coverage (Klindworth et al., 2013). These primers included a 10-base unique barcode to identify each sample (on forward primer only) and GS FLX Titanium Primers. The resulting composite forward primer was 5′- *CCA TCTCATCCCTGCGTGTCTCCGACTCAG*NNNNNNNNNN**CCTACGGGNGGCWGC A**G-3′, where the italicized sequence is the GF FLX Titanium Primer A, the NNNNNNNNNN sequence corresponds to the unique 10-base barcode, and the bold sequence is the universal primer S-D-Bact-0341-b-S-17. The composite reverse primer was the same for all amplifications: 5′-*CCT ATCCCCTGTGTGCCTTGGCAGTCTCAG***GACTACHVGGGTATCTAATCC -3**′, where the italicized sequence is the GF FLX Titanium Primer B and the bold sequence is the universal primer S-D-Bact-0785-a-A-21.

PCR amplification of 16S rRNA was performed in duplicate using a Veriti™ 96-well thermal cycler (Applied Biosystems, Foster City, CA, USA) in a 50-μL final volume containing 0.5 μM forward and reverse primers, a 5 μL DNA sample and 1 × NEBNext High Fidelity PCR Mastermix (New England Biolabs, Evry, France). The PCR conditions were as follows: denaturation step at 95°C for 5 min, followed by eight cycles of denaturation at 98°C for 10 s, annealing at 61°C for 30 s, and extension at 72°C for 30 s and 22 cycles of denaturation at 98°C for 10 s and extension at 72°C for 30 s. A final extension step was performed for 5 min at 72°C. Blank controls, in which no DNA was added to the reaction, were performed. Amplicon quality was checked on 1% agarose gel in 0.5X TBE. No amplicons were visible with blank control. Amplicons were purified with Agencourt AMPur XP magnetic beads (Beckman-Coulter) and quantified using the Quant-iT PicoGreen dsDNA Assay Kit (Invitrogen).

Pyrosequencing was performed on a GS FLX system (454/Roche) at the “Functional and Environmental Genomics” platform (OSUR, Rennes, France). DNA sequence datasets are available at the European Nucleotide Archive, under the accession number PRJEB12570.

### Sequence library analysis

Data quality control and analyses were performed using the QIIME pipeline (Caporaso et al., 2010) hosted on the INRA MIGALE bioinformatics platform. Reads were first assigned to samples using the split_library.py script. This step also included a quality filtering step based on read length (200 < read length < 1000 bp) and quality: Number of ambiguous bases < 6; Mean quality score > 25; Maximum homopolymer < 6; No mismatches in primers). Clustering and taxonomy assignment were then done using the pick_de_novo_otus.py script. Briefly, reads were clustered using UCLUST with a degree of similarity of 97% to generate OTUs. Taxonomy was then assigned using the Ribosomal Database Project (RDP) Classifier and the Greengenes 16S reference database (available at: http://blog.qiime.org, version available on the server in July 2014). This step generated the table of abundance for each sample (i.e., the proportion of reads corresponding to a given taxon with regard to the total number of reads of a given sample). Tables of abundance were available at different taxonomic levels: phylum (L2), class (L3), order (L4), family (L5), genus (L6).

In order to delve deeper into the taxonomic identification of discriminant genera (see below, Section Statistical Analysis), data were further analyzed by combining three taxonomy assignment methods (Blast, RDP, and Uclust) embedded in the QIIME pipeline (Caporaso et al., 2010). When the deepest RDP taxonomy assignment was at genus level, it was compared with Blast and Uclust assignment result. If Blast or Uclust assigned a deepest classification, it was retained.

Alpha diversity, which is the diversity within samples, was determined through the estimation of the Shannon index using the alpha_rarefaction.py script. The Shannon index is a widely used nonparametric index of alpha-diversity that measures richness (the total number of OTUs) and evenness (the relative abundance of OTUs). More information on the Shannon index formula can be obtained from qiime documentation (http://qiime.org/scripts/alpha_diversity.html). The Shannon index was calculated from 10 sequences onwards, with a step of 297 sequences, as automatically determined by the script.

Beta diversity, which is the diversity between samples, was estimated through the measurement of the weighted UniFrac distance, followed by Principal Component Analysis (PCA) using beta_diversity_through_plots.py script. This non-taxonomic analysis allowed to analyse diversity between samples based on the relative abundance of each OTU within the samples.

### Statistical analysis

Taxonomic profile analysis was first done by considering the abundance of the dominant genera, which means that they were present in at least 5‰ abundance in a given sample. Genera present in less than 5‰ were also considered but they were pooled in the corresponding phylum and referred to as “phylum_others.” These data, corresponding to dominant genera, are available in Table S4. A clustering of samples was then undertaken by performing a PCA on these dominant genera, followed by a hierarchical clustering using R software (R Development Core Team, 2013). This clustering made it possible to cluster samples into two groups.

Taxonomic profiles were then subjected to differential analysis by the use of the linear discriminant analysis (LDA) effect size (LEfSe) method (available at http://huttenhower.sph.harvard.edu/lefse/) in order to identify taxa that were discriminant between the two clusters (Segata et al., 2011). For this analysis, a table of taxa abundance including all the different taxonomic levels was used. Briefly, the first step of the LEfSe method analyzed all taxonomic units, testing whether abundance in the different clusters (designed as classes by the LEfSe method) are differentially distributed, using a Kruskal-Wallis rank sum test. An LDA model was then built to estimate the effect size of each differentially abundant taxon, i.e., to rank the differentially abundant taxa according to their relative difference among classes/clusters. This step resulted in a list of taxonomic units that are discriminative with respect to the classes/clusters and ranked according to the effect size with which they differentiate clusters.

Statistical analysis was also performed on alpha diversity (Shannon index) between groups of quarters with different health status using a Mann Whitney test with a *p* < 0.05.

### Determination of total bacterial population of samples

The total bacterial population of samples was estimated by quantitative PCR on 16S rRNA using primers S-D-Bact-0341-b-S-17 and S-D-Bact-0785-a-A-21. Quantitative PCR was performed using a CFX96 Touch real-time system (Bio-Rad, Hercules, CA, USA). The reaction mixture contained SsoAdvanced Universal SYBR Green Supermix (Biorad), 0.5 μM of each primer and 5 μL of total genomic DNA samples. Thermal cycling consisted of 30 s at 95°C, followed by 40 cycles of 10 s at 95°C, and 30 s at 60°C. Genomic DNA of *Staphylococcus aureus* was used to generate standard curves. Data were normalized with regard to the volume of foremilk and are thus expressed as equivalent copy numbers of *S. aureus* genomes per ml of foremilk.

## Results

### Sequencing results and alpha diversity analysis

In the present study, the teat microbiome was investigated on 31 quarters with different history of mastitis but free of inflammation at sampling time. SCC was lower than 50,000 cells/ml in all quarters retained for the analysis except one, namely V4 ARG (84,000 cells/ml). Sequencing analysis resulted in a total of 122,977 reads with an average length of 416.8 bp that passed quality filters and that were assigned to bovine teat microbiome samples. Statistics regarding sequencing and filtering steps are presented in Table S2. An average number of 3967 reads per sample was obtained. Sequencing of the negative control (PCR without genomic DNA) resulted in 125 reads, which were not further analyzed.

**Figure 1:**
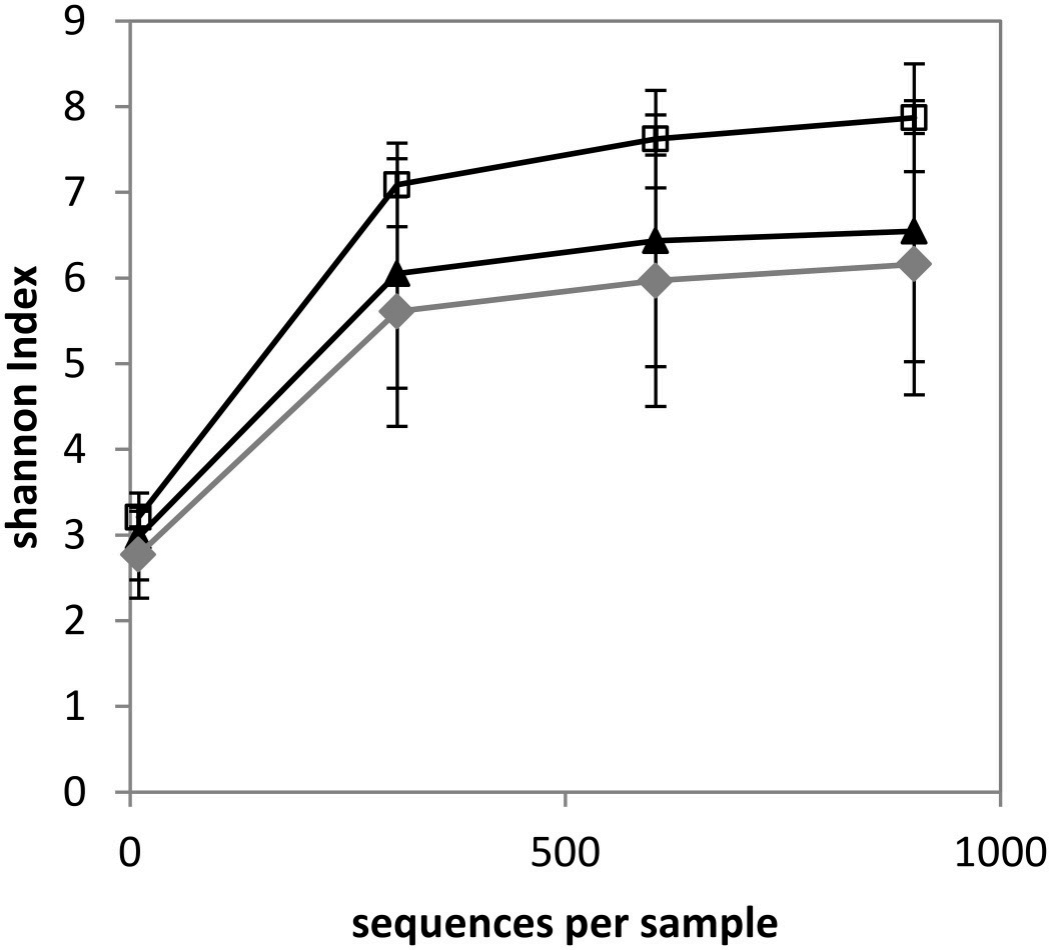
**Rarefaction curves of samples with regard to quarter status as determined by the Shannon index**. White squares, healthy quarters (Hq); gray diamonds, not determined status (NDq); black triangles, quarters that have already developed mastitis (Mq).

Alpha diversity was estimated through the measurement of the Shannon index, which combines richness and evenness. Rarefaction curves were shown to flatten for each sample, indicating that sequencing was deep enough to estimate the microbiome composition (data not shown). Rarefaction curves were also determined according to quarter status (Figure 1). The Shannon index of Hq quarters was significantly higher compared to Mq quarters for a number of sequences per sample ≥307 and it was significantly higher compared to NDq quarters for a number of sequences per sample ≥10 (as determined using a Mann-Whitney Test with *p* < 0.05). As an illustration, the Shannon indices reached 7.87, 6.54, and 6.16 (for 901 sequences), for Hq, Mq, and NDq quarters, respectively. No significant differences were observed between Mq and NDq quarters.

### Taxonomic profile analysis

**Figure 2:**
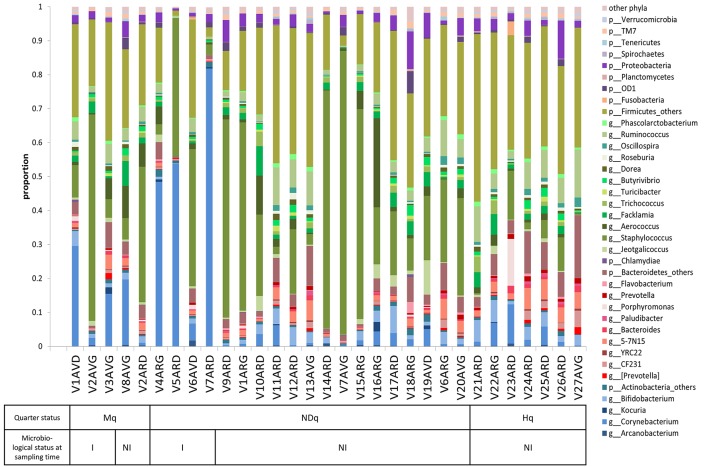
**Bovine teat taxonomic profiles combining taxonomic levels L2 (phyla) and L6 (genera)**. Each bar represents a sample. Genera present in at least 5‰ abundance in a given sample are displayed, whereas genera present in less than 5‰ are pooled in the corresponding phylum and referred to as “phylum_others.” Actinobacteria are displayed in blue, Bacteroidetes in red, Firmicutes in green and Proteobacteria in purple. Samples were characterized with regard to: (i) quarter health status (Mq, has already developed mastitis; Hq, healthy; NDq, not determined status); and (ii) microbiological status at sampling time (I, infected; NI, not infected), as a result of plate counts on Columbia II containing 5% sheep blood.

Assignment of reads to taxonomic units led to the determination of taxonomic profiles of the bovine teat microbiome for quarters with different histories regarding mastitis. The relative abundance of each taxonomic unit, from phylum to genus, is presented for each sample in Table S3. Visualization of the taxonomic profile is proposed on Figure 2, which combines abundance tables at the phylum (L2) and genus (L6) levels: only dominant genera whose abundance was higher than 5‰ in at least one sample were included in Figure 2, whereas other genera were pooled in the corresponding phylum (see Table S4 for the corresponding abundance table combining L2 and L6 levels). An overview of Figure 2 indicates that the taxonomic profile is dominated by genera belonging to the Firmicutes, followed by genera belonging to Bacteroidetes, Actinobacteria, and to a lesser extent, Proteobacteria. Median abundances of these four phyla are 70.1, 8.1, 7.3, and 2.5%, respectively. Taking a more in-depth look, dominant genera included *Staphylococcus* (with an average abundance of 23.8%), *Corynebacterium* (10.1%), *Ruminococcus* (4.9%), *Aerococcus* (3.2%), *Bifidobacterium* (2.6%), *Flacklamia* (2.4%), *Jeotgalicoccus* (1.3%), *Trichococcus* (1.2%), and *Oscillospira* (0.8%), as well as several Lachnospiraceae such as *Butyrivibrio, Dorea*, and *Roseburia*, and two genera belonging to Bacteroidetes, namely *Bacteroides* and *Prevotella*, which all exhibited an average abundance of around 0.5%. Comparison of taxonomic profiles revealed high variability between samples. Some Mq and NDq samples were clearly dominated by one genus, notably *Staphylococcus* and *Corynebacterium*, while others, notably among Hq and NDq quarters, showed a more balanced profile. NDq taxonomic profiles were highly variable, with a continuum of profiles ranging from some profiles resembling those of Hq samples to some closer to those of Mq samples.

Taxonomic profile analysis makes it possible to determine the relative abundance of taxonomic units but not the absolute amount of these taxonomic units within the ecosystem. In order to check whether taxonomic profile variability could be related to the variation of the total population of the microbiota, the total population was determined by performing quantitative PCR on total genomic DNA using the universal primers S-D-Bact-0341-b-S-17 and S-D-Bact-0785-a-A-21 that were used to generate our 16S rRNA amplicons. Absolute quantification was performed taking the *S. aureus* genome as a standard. The median population was found to be 4.76, 4.25, and 4.6 log_10_ (*S. aureus* genome equivalent copy number) per mL of foremilk for Mq, NDq and Hq quarters respectively, indicating no major change in the total bacterial population inside the teat.

### Clustering of samples

**Figure 3:**
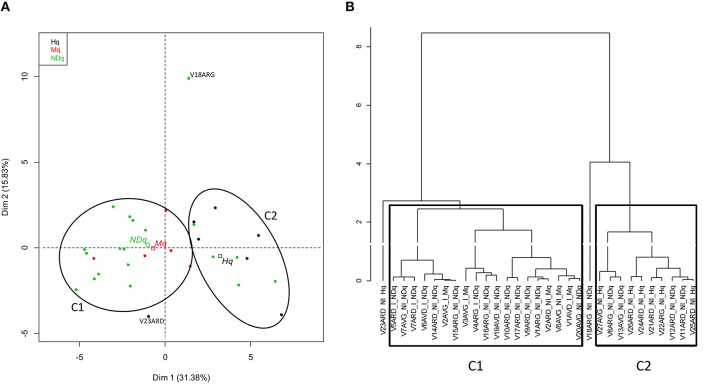
**Principal Component Analysis and hierarchical clustering on bovine teat taxonomic profiles**. PCA was performed on an abundance table combining taxonomic levels L2 and L6. **(A)** Individual factor map. Samples are indicated by points and colored with regard to quarter health status. Centroid positions are indicated by squares for quarter status (Hq, Mq, NDq); **(B)** Hierarchical clustering of samples according to ACP results. All samples but one (V23ARD), classified as healthy quarters (Hq), are clustered together (C2), whereas all samples that have already developed mastitis (Mq) and those that display an infected microbiological status (I) are clustered together (C1).

Comparison of bovine teat taxonomic profiles was further achieved by performing a Principal Component Analysis on the L2-L6 abundance table, followed by a hierarchical clustering (Figure 3). Good separation between Hq and Mq quarters was obtained, mainly on the first dimension, where the first two dimensions were responsible for 47% of the total variance (Figure 3A). NDq quarter distribution revealed an overlap with Hq and Mq quarter distribution. However, the centroid of the NDq quarters was closer to that of the Mq quarters. Separation between Hq and Mq quarters was also established through the hierarchical clustering. Setting the threshold for clustering at 2.6, they fell into two separate clusters, referred to as C1 and C2 on Figure 3B. Hence, all Mq quarters were clustered together into Cluster C1. This cluster also included several NDq quarters. Mq quarters did not cluster into a single sub-cluster within C1 but were instead distributed among several sub-clusters together with NDq quarters. Similarly, all Hq quarters but one (V23ARD) were clustered together in Cluster C2, together with a few NDq quarters.

Diversity between samples was also analyzed considering OTU distribution rather than taxonomic profiles, by measuring weighted Unifrac distances between samples. As observed using taxonomic profiles, Hq and Mq quarters were separated whereas NDq quarters distribution revealed an overlap with Hq and Mq quarter distribution (Figure S1). Clusters C1 and C2, which resulted from clustering based on taxonomic profiles (see above) are indicated on Figure S1.

### Discriminant analysis

**Figure 4:**
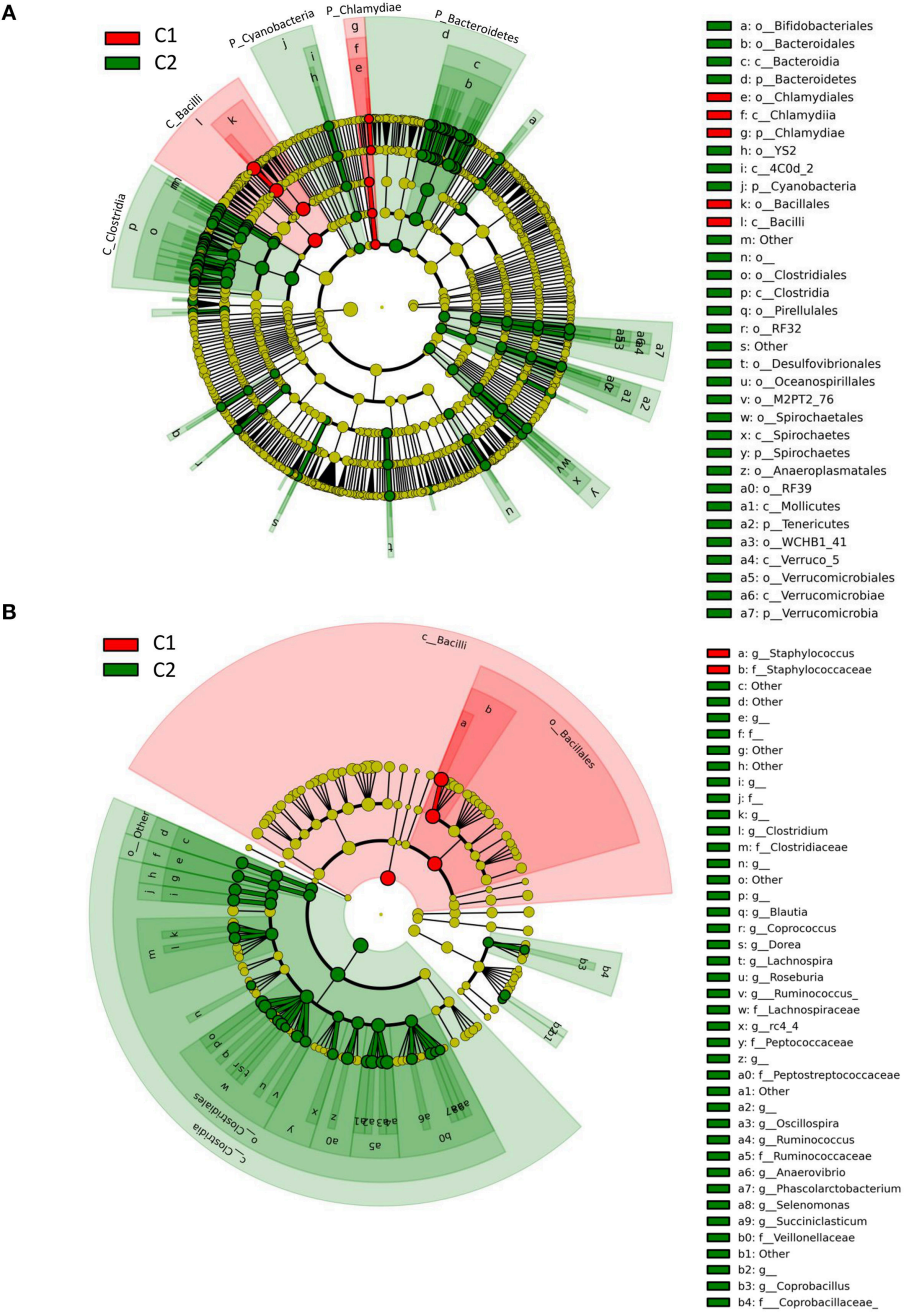
**Taxonomic representation of differentially abundant taxa between Cluster 1 (contains quarters susceptible to mastitis) and Cluster 2 (contains healthy quarters), as determined by the LEfSe pipeline**. Differences are represented by the color of the cluster where the taxon is more abundant (red indicates Cluster 1 and green indicates Cluster 2). The diameter of each circle is proportional to the abundance of the taxon. **(A)** Root of the cladogram refers to Bacteria. Only taxonomic levels L2 (phylum) to L4 (order) are labeled. See Table S5 for taxonomic level L5 (family) and L6 (genera). **(B)** Detailed composition of bacteria belonging to Bacilli and Clostridia classes shown in **A** (root of the cladogram refers to the phylum Firmicutes).

Data were subjected to discriminant analysis in order to identify differentially abundant taxonomic units between Clusters C1 and C2 (Figure 4, Table 1). Complete results of discriminant analysis, corresponding to the LDA score and *p*-value, are included in Table S5. An overview of the cladogram indicated that most of the discriminant taxa were more abundant in Cluster C2 (Figure 4A). Among the most discriminant taxa with an LDA score higher than 5, the Bacilli class was found to be more abundant in Cluster C1 than in Cluster C2, with a mean relative abundance of 52.6 and 22.7%, respectively. This was notably related to a higher abundance of *Staphylococcus*, which belongs to Bacilli, and which was present at 34 and 6.7% in Clusters C1 and C2, respectively (Figure S2). Investigating in more depth the taxonomic identification of this discriminant taxon made it possible to identify ~13% of total *Staphylococcus* reads at the species level, which mainly corresponded to *S. aureus* and *S. equorum* (data not shown). For two quarters, namely V1ARG and V5ARD (attributed to the C1 cluster), which were dominated by *Staphylococcus*, 28 and 30% of *Staphylococcus* reads were assigned to *S. aureus*. For the seven other quarters (also attributed to the C1 cluster) that had a high abundance of *Staphylococcus*, reads assigned to *S. aureus* corresponded to 0.4–2.2% of *Staphylococcus* reads.

**Table 1:** **Differentially abundant genera between Cluster 1 (contains Mq quarters) and Cluster 2 (contains Hq quarters) as determined by the LEfSe pipeline**.

**Taxonomic unit**		**Average abundance**		**Median abundance**		**Standard deviation**		**LDA score**	**pval**
**Phylum**	**Genera**	**C1**	**C2**	**C1**	**C2**	**C1**	**C2**		
**GENERA MORE ABUNDANT IN CLUSTER 1**									
Firmicutes	**Staphylococcus**	**33.97**	**6.70**	**28.62**	**2.62**	**25.16**	**8.52**	**5.12**	**0.0015**
**GENERA MORE ABUNDANT IN CLUSTER 2**									
Euryarchaeota	Methanocorpusculum	0.00	0.02	0.00	0.00	0.00	0.05	3.07	0.0134
Actinobacteria	**Bifidobacterium**	**1.91**	**4.23**	**1.54**	**3.99**	**1.85**	**1.48**	**4.01**	**0.0010**
	[Prevotella]	0.23	0.72	0.07	0.62	0.40	0.59	3.40	0.0010
	CF231	0.37	1.79	0.32	1.77	0.32	1.36	3.87	0.0010
	YRC22	0.11	0.34	0.10	0.38	0.12	0.18	3.11	0.0076
	**5-7N15**	**1.32**	**4.46**	**1.21**	**4.72**	**1.12**	**1.61**	**4.20**	**0.0001**
	Bacteroides	0.22	0.92	0.12	0.86	0.24	0.49	3.57	0.0002
	Paludibacter	0.18	0.57	0.11	0.61	0.17	0.45	3.35	0.0116
	Parabacteroides	0.02	0.09	0.00	0.08	0.04	0.07	2.86	0.0015
	Prevotella	0.26	1.00	0.16	0.89	0.32	0.35	3.57	0.0001
	Hymenobacter	0.00	0.01	0.00	0.00	0.00	0.01	2.78	0.0473
Firmicutes	Leuconostoc	0.01	0.04	0.00	0.03	0.03	0.04	2.76	0.0265
	Turicibacter	0.30	0.65	0.23	0.45	0.29	0.52	3.22	0.0346
	Clostridium	0.16	0.50	0.12	0.36	0.11	0.37	3.23	0.0004
	[Ruminococcus]	0.13	0.24	0.09	0.20	0.12	0.12	2.78	0.0059
	Blautia	0.08	0.20	0.05	0.17	0.08	0.12	2.88	0.0043
	Butyrivibrio	0.95	1.54	0.74	1.36	0.70	0.82	3.46	0.0435
	Coprococcus	0.07	0.25	0.05	0.20	0.08	0.15	2.99	0.0003
	Dorea	0.54	1.10	0.48	0.99	0.39	0.45	3.39	0.0033
	Lachnospira	0.01	0.03	0.00	0.01	0.01	0.03	3.16	0.0360
	Roseburia	0.28	0.89	0.20	0.81	0.34	0.41	3.50	0.0002
	rc4-4	0.08	0.27	0.03	0.21	0.15	0.22	3.00	0.0020
	Oscillospira	0.48	1.61	0.44	1.50	0.40	0.64	3.75	0.0001
	**Ruminococcus**	**2.80**	**9.21**	**2.67**	**9.12**	**1.69**	**2.61**	**4.49**	**0.0000**
	Anaerovibrio	0.05	0.42	0.02	0.34	0.06	0.46	3.28	0.0002
	Dialister	0.00	0.02	0.00	0.00	0.00	0.04	2.51	0.0473
	Phascolarctobacterium	0.43	1.23	0.37	1.26	0.39	0.25	3.59	0.0002
	Selenomonas	0.01	0.06	0.00	0.04	0.02	0.05	3.13	0.0005
	Succiniclasticum	0.02	0.05	0.00	0.03	0.05	0.07	2.69	0.0092
	Coprobacillus	0.01	0.03	0.00	0.04	0.04	0.02	3.00	0.0057
Proteobacteria	Rhodoplanes	0.00	0.01	0.00	0.00	0.00	0.02	2.94	0.0473
	Sphingopyxis	0.00	0.01	0.00	0.00	0.00	0.01	3.06	0.0473
	Desulfovibrio	0.07	0.20	0.06	0.15	0.05	0.17	2.88	0.0088
	Arcobacter	0.00	0.03	0.00	0.00	0.01	0.05	2.58	0.0178
	Marinospirillum	0.05	0.20	0.00	0.19	0.10	0.14	2.95	0.0027
Spirochaetes	Treponema	0.04	0.32	0.02	0.26	0.05	0.26	3.17	0.0009
Tenericutes	RFN20	0.00	0.01	0.00	0.00	0.00	0.02	2.97	0.0473
Verrucomicrobia	Akkermansia	0.10	0.35	0.06	0.31	0.12	0.16	3.12	0.0003

*Average and median abundances (expressed as percentages) as well as standard deviation are presented for each cluster. LEfSe results include LDA score and pval. The most discriminant genera (LDA score > 4) are in bold*.

Conversely, the Clostridia class was the most discriminant taxonomic unit (LDA score > 5), whose abundance was higher in Cluster C2 (containing healthy quarters) than in Cluster C1 (41.9 and 16.3% in C2 and C1, respectively). This was related to a higher abundance of genera belonging to the Lachnospiraceae family such as *Butyrivibrio, Dorea, Roseburia, Coprococcus, Blautia* and *Lachnospira*, and genera belonging to the Ruminococcaceae family such as *Ruminococcus* and *Oscillospira*. Among these genera, *Ruminococcus* was present in C2 and C1 with a mean abundance of 9.1 and 2.8%, respectively. Other major discriminant taxa (LDA score > 4), whose abundance was significantly higher in Cluster C2 than in Cluster C1, included the Bifidobacteriales order and notably *Bifidobacterium* whose abundance was 1.9 and 4.2% in C1 and C2, respectively. Likewise, a higher abundance of Bacteroidetes was observed in C2 compared to C1 (relative abundance of 18.6 and 6.1% in C2 and C1, respectively). This was mainly related to a higher abundance of the Bacteroidales order. Other discriminant taxonomic units with lower potential of discrimination are listed in Table S5 and include taxa belonging to Proteobacteria, several subdominant phyla such as Cyanobacteria, Spirochaetes, Tenericutes, and Verrucomicrobia, which were more abundant in Cluster C2 than in Cluster C1, and Chlamydiae, which was more abundant in Cluster C1 than in Cluster C2.

## Discussion

For years now, mammary gland inflammation has been considered as the result of host pathogen interaction, a result that depends notably on bacterial and host genetic determinants (Burvenich et al., 2003; Le Marechal et al., 2011). Metagenomic exploration of several ecosystems, has revealed not only their richness and complexity but the link between their composition and ecosystem or organ functionality and health (Ravel et al., 2011; Evans et al., 2013; Belkaid and Segre, 2014; Schokker et al., 2015; Weimer, 2015). We could then wonder whether the microbiota associated with the bovine mammary gland can be related to the mammary gland health status. In this study, we investigated the composition of the bovine mammary gland microbiota in relation to quarter history with regard to mastitis using a marker gene analysis (pyrosequencing on 16S V3-V4 region). Contrary to previous studies that focus on changes in microbiome composition during mastitis (Bhatt et al., 2012; Oikonomou et al., 2012, 2014; Kuehn et al., 2013), we explored the bovine mammary gland microbiota, apart from the infectious episode, taking the animal's history rather than its health status at the time of sampling into account. In addition, contrary to these previous studies that focused on the milk microbiome, we targeted the internal teat microbiota. Infectious cycles generally start with ascending colonization of the mammary gland through the teat toward the cistern. Microbiota associated with teat canal and teat cistern epithelium may thus constitute a first microbiological barrier that can compete with pathogens. Such barrier effects were previously reported in other contexts such as gut or vaginal ecosystems (Martin, 2012; Macfarlane, 2014).

Bovine mammary microbiome analysis revealed strong variations between quarters, with some quarters clearly dominated by one taxonomic unit, whereas others displayed a more balanced profile. Of note, taxonomic profiles of quarters corresponding to the same animal were not necessarily more similar than those of quarters corresponding to different animals, as illustrated for quarters V7ARD and V7AVG, which corresponded to the same animal (V7) but did not belong to the same cluster based on their taxonomic profiles. Changes in taxonomic profiles were not related to major variations of the microbiota total population, which was similar in the three groups of quarters (Hq, Mq and NDq). Taxonomic profiles were dominated by Firmicutes in most cases, followed by three other phyla, namely Bacteroidetes, Actinobacteria, and Proteobacteria. Most of the dominant genera presented in Figure 2 have been described as belonging to bovine mammary microbiota, isolated from teat apices or milk, and characterized either by DGGE or 16S rRNA pyrosequencing (Braem et al., 2012, 2013; Oikonomou et al., 2012, 2014; Zhang et al., 2015). In particular, *Staphylococcus* is among the dominant genera in several studies on bovine milk as well as human milk (Hunt et al., 2011; Braem et al., 2012; Oikonomou et al., 2014). Nevertheless, some discrepancies exist since the relative abundance of these genera varies between the different studies. For instance, *Propionibacterium* and *Aeribacillus* were the dominant genera in healthy quarters of Oikonomou's study (~10% average abundance; Oikonomou et al., 2012), whereas they were poorly present in our study (average abundance lower than 0.5%). Likewise, *Streptococcus* was among the most prevalent genera, regardless of the quarter status in Oikonomou's report (Oikonomou et al., 2014), whereas *Streptococcus* mean abundance was below 0.04% in our study. Several explanations may account for such discrepancies, including the sampling itself and technical parameters such as the method of bacterial lysis and genomic DNA extraction and the 16S rRNA variable region retained for amplification. Of note, DNA extraction was performed on a bacterial pellet washed and treated by triton to lyse bovine cells and remove contaminating bovine DNA. We cannot totally exclude that these separation steps led to the elimination or killing of some bacterial species, although the total bacterial population was not altered. The impact of DNA extraction methods and sampling techniques has been clearly demonstrated on the microbial community composition of cow and sheep rumen (Henderson et al., 2013). In addition, microbiota has already been found to vary between herd and geographical areas (Espeche et al., 2012). Data analysis can also introduce some bias. For instance, in this study, sequences have not been checked for chimeras. Chimeras have been shown to have minor influence on results generated by 454 pyrosequencing (removing of less than 1% of reads), whereas denoising pipelines such as ChimeraSlayer can introduce changes to the reads, thus questioning the relevance of these denoising pipelines (Gaspar and Thomas, 2013).

### Variations of the bovine teat microbiome correlated with mastitis history

Firstly, analysis of taxonomic profile diversity revealed that alpha diversity significantly varied with regard to the quarter's status. Healthy quarters exhibited a significantly higher diversity than quarters that had already undergone clinical mastitis, as revealed by the Shannon diversity index of 7.87 and 6.54 for Hq and Mq quarters, respectively. Such changes of diversity in relation to health status have already been observed by Braem et al. (2012), who reported a higher number of genera in the teat apex from non-infected quarters, as determined by DGGE. Likewise, a lower diversity has been reported in the uterine microbiota of cows suffering from metritis compared to healthy animals, as revealed by DNA pyrosequencing (Santos and Bicalho, 2012).

Secondly, clustering of taxonomic profiles made it possible to separate all heathy quarters but one (V23ARD) from those that had already undergone clinical mastitis. Of note, analysis of diversity between samples based on OTU distribution was in agreement with clustering obtained using taxonomic profiles. In particular, separation between Hq and Mq quarters was obtained. The sample V23ARD was more closely related to the other Hq quarters when considering OTU distribution. It should be noticed here that, in the lack of SCC recording at quarter level on the previous lactations, assignment of quarters to Hq was based on SCC recording on composite milk. However, we could not totally exclude, by using a threshold lower than 250,000 cell/mL on composite milk, that some Hq quarters had previously developed subclinical mastitis. Indeed, in a previously published meta-analysis, the mean SCC was 68,000 cells/ml in bacteriologically negative quarters, whereas it was higher than 105,000 cells/ml in quarters that harbored intra-mammary infections (Djabri et al., 2002). The likehood that cows with composite milk SCC between 100,000 and 250,000 cells/mL had at least one inflamed quarter was not negligible. Nevertheless, despite the above-mentioned reservations on our criteria, almost all Hq quarters clustered together and were separated from Mq quarters.

One Hq quarter was isolated, namely V23ARD, which contained high abundances of *Porphyromonas* and Fusobacteria compared to all the other samples (Figure 2). *Porphyromonas levii* and *Fusobacterium necrophorum* have been detected in most of the mastitic milk samples by Oikonomou and coworkers, although they were not directly responsible for the mastitis (Oikonomou et al., 2012). Both species, *F. necrophorum* and *P. levii*, have been shown to be involved in summer mastitis, acting in synergy with other pathogens such as *Trueperella pyogenes* (Pyörälä et al., 1992). Interestingly, this animal (V23) exhibited a moderate increase of SCC on composite milk (>300,000 cells/mL on milk collected from the four quarters) at the end of the analyzed lactation. Whether this SCC increase was related to a subclinical mastitis on this quarter at the end of lactation was however not determined.

NDq quarters clustered either with Mq quarters in Cluster C1 or Hq quarters in Cluster C2. The clustering of most NDq quarters with Mq quarters strongly suggests that several NDq quarters, but not all, have a history of subclinical mastitis. Of note, all NDq quarters that were infected but not inflamed at the time of sampling were included in C1. However, we cannot totally exclude that the presence of pathogens in cistern milk sample was due to contamination by pathogens present in the teat cistern or teat canal and washed out during milking.

Variation of the taxonomic profile in relation to heath status has already been reported in the mammary gland and uterine contexts (Santos et al., 2011; Braem et al., 2012; Oikonomou et al., 2012, 2014; Santos and Bicalho, 2012; Kuehn et al., 2013). However, all these studies focused on changes in the bacterial community at the time of inflammation. Here, we showed that the microbiota composition of quarters with different health statuses was also altered apart from the infectious episode (of note, animals had not received antibiotic treatment for the last 2 months at least). These different taxonomic profiles may result from an infectious episode and or antibiotic treatment. Alternatively, we cannot exclude that taxonomic profiles were already altered prior to the infection and that these alterations contributed to the infection.

### Discriminant analysis revealed taxonomic markers of a quarter's health status

Discriminant analysis between Cluster C1, which includes all Mq quarters, and Cluster C2, which includes all Hq quarters but one, led to the identification of taxonomic markers of these two clusters. Discrimination occurred at different taxonomic levels. Most taxonomic markers belong to Firmicutes and exhibited a higher abundance in C2. The most discriminant taxa were also among the most abundant ones. Hence, within Firmicutes, Bacilli were significantly more abundant in C1 than in C2, with average abundance of 52.6 and 22.7%, respectively, whereas Clostridia were significantly more abundant in C2 than in C1, with average abundances of 41.9 and 16.3% in C2 and C1, respectively. Other discriminant taxa included *Bifidobacterium* and the phylum Bacteroidetes, whose average abundances were 2-fold and 3-fold higher in C2 quarters than in C1, respectively. The origin of these differential taxonomic profiles and, notably, the change in the Bacilli/Clostridia ratio remain to be elucidated. Several of the genera belonging to Clostridia as well as to Bacteroidetes are common with the rumen microbiome (Jami and Mizrahi, 2012; McCann et al., 2014; Sandri et al., 2014; Kumar et al., 2015), suggesting a transfer between the rumen to the mammary gland, possibly in a way similar to the gut-breast axis observed from mother to neonate in humans (Jost et al., 2014) but more probably by ascending colonization through the teat canal (fecal contamination).

The high abundance of Bacilli in C1 is notably related to a high amount of *Staphylococcus*, whose abundance was 34% on average in C1 (vs. 6.7% in C2) and even reached 83% in the quarter V7AVG. Two of the nine quarters dominated by *Staphylococcus*, namely V1ARG and V5ARD, had ~30% of *Staphylococcus* reads assigned to *S. aureus*. The seven other quarters had only 0.4–2.2% of *Staphylococcus* reads assigned to *S. aureus*, indicating that other and possibly multiple *Staphylococcus* species were responsible for this profile. *S. aureus* and coagulase negative staphylococci (CNS) are among the main etiologic agents involved in mastitis (Dufour et al., 2012; Keane et al., 2013, p. 2). *S. aureus* is considered as a major pathogen in bovine mastitis, together with *Streptococcus uberis* and *Escherichia coli*, while CNS are considered as minor pathogens, leading to milder inflammations and subclinical mastitis. The Staphylococci reservoir is mainly the mammary gland, whereas *E. coli* is considered as an environmental pathogen. The *S. uberis* reservoir includes the environment and the mammary gland. *E. coli* generally rapidly leads to acute infections followed by resolution, while *S. aureus* and *S. uberis* are likely to persist within the mammary gland. In agreement, we found Staphylococci in all quarters, whereas *E. coli* was not detected and the *Streptococcus* sp. mean abundance was 0.04%. Although, it may be speculative since we do not know which pathogen was involved in the recorded SCC increases, the higher abundance of Staphylococci in C1 quarters may have favored their ability to reach the cistern through ascending colonization, thus provoking inflammation. It may also have favored the emergence of other pathogens through the interaction with the immune system. Such “collaboration” between pathogens has already been observed, i.e., in the bovine genital tract context. Hence, metritis often starts by contamination of the uterine lumen by *E. coli*, followed by post-infection by other species such as *Arcanobacterium pyogenes* or *Fusobacterium necrophorum* (Williams et al., 2007). Interestingly, *Corynebacterium* has also been previously associated with intra-mammary infections, but as a colonizer of the teat canal rather than a causal agent of mastitis (Braem et al., 2012). Sam Ma et al. (2015) have recently suggested a potential ‘evil’ alliance of *Staphylococcus* and *Corynebacterium* in the human milk microbiome against the benign microbiota, leading to dysbiosis and enabling mastitis (Sam Ma et al., 2015). *Corynebacterium* was not discriminant between C1 and C2 due to strong variations of its abundance between quarters. However, six quarters belonging to C1, namely V1AVD, V3AVG, V4ARG, V5ARD, V7ARD, and V8AVG, exhibited a high abundance of *Corynebacterium*, from 15 to 82%, whereas the mean abundance of *Corynebacterium* in C2 quarters was much lower at 2.4%.

## Concluding remarks

In this study, we showed that the bovine teat bacterial community varies in relation to animal history regarding mastitis, apart from the infectious episode. Hence, healthy quarters and those that had already developed mastitis exhibited divergent taxonomic profiles that could be separated into two clusters. Discriminant analysis made it possible to identify taxonomic markers of these two clusters, which could become good candidates to develop diagnostic tools of mammary gland health. However, in this study, the exploration of bovine mammary microbiota was done on one herd. Extending this work to other herds will help to test the robustness of these taxonomic markers, to adjust them or define new ones. Several parameters probably influence microbiota, and should be investigated, including diet, herd management, animal housing, milking number, hygienic procedures during milking, or animal genetics and lactation number. Of note, the average number of lactations was not statistically different in clusters C1 and C2 (2.63 and 2.5 in C1 and C2 respectively) nor in quarter groups (average number of lactations of 2.60, 2.55 and 2.62 for Mq, NDq, and Hq quarters respectively). However, only multiparous animals were included in the study, mainly in the second and third lactations.

The existence of differential taxonomic profiles between C2 and C1 questions whether mammary gland microbiota could interfere with host pathogen interaction. However, investigating the dynamics of this bacterial community all along the lactations, starting on animals prior to any mammary gland infection, will be necessary to determine whether differences of the bacterial community in C1 contributes to or results from mastitis development, or both. Additional investigations will also be necessary to investigate the functionality of this microbiota: are these bacteria alive? How do they interact with mammary gland epithelium? At the very least, these results encourage us to take the teat microbiota into account in bovine mammary gland health management and to explore strategies that would preserve or restore a balanced microbiota as a prophylactic measure.

## Author contributions

Substantial contributions to the conception or design of the work: SE, YL, PM; acquisition: LR, DB, JL, PL, JA, SE; analysis and interpretation of data for the study: SE, HF, AN. Drafting of the study: SE, HF; revising it critically for important intellectual content: all of the authors. All of the authors approved the final version of the manuscript and agreed to be accountable for all aspects of the work by ensuring that questions related to the accuracy or integrity of any part of the work are appropriately investigated and resolved.

## Funding

This work was financially supported by the INRA-funded MEM project (Milk Ecosystem and Udder Health).

### Conflict of interest statement

The authors declare that the research was conducted in the absence of any commercial or financial relationships that could be construed as a potential conflict of interest.

## Supplementary material

Figure S1 **Principal Component Analysis based on weighted UniFrac distances**. Samples are indicated by points and colored with regard to quarter health status: Hq, (blue); Mq, (red); NDq, (green).

Figure S2 **Selected boxplot of taxonomic units that are differentially abundant between Cluster 1 (contains quarters susceptible to mastitis) and Cluster 2 (contains healthy quarters) as determined by the LEfSe pipeline**. The box represents the 75% (upper) and 25% (lower) quartiles and the black line inside the box represents the median. Abundance of the classes Bacilli **(A)** and Clostridia **(B)** the phylum Bacteroidetes **(C)** and the genera *Staphylococcus* **(D)** *Ruminococcus* **(E)** and *Bifidobacterium* **(F)**.

Table S1 **Quarter status based on the history of the animal**.

Table S2 **Sequencing overview and filtering statistics**. Reads were filtered with regard to their length and quality and assigned to samples using the split_library.py script of QIIME.

Table S3 **Bovine teat taxonomic profiles combining all taxonomic levels (from phylum to genera)**. The abundance of each taxonomic unit is presented for each sample, completed by the average and median abundances for each cluster (columns AN-AS). Samples were characterized with regard to: (i) quarter health status (Mq, has already developed mastitis; Hq, healthy (no mastitis at all); NDq, unknown status), and (ii) microbiological status at sampling time (I, infected; NI, not infected).

Table S4 **Bovine teat taxonomic profiles combining taxonomic levels L2 (phyla) and L6 (genera)**. Genera present in at least 5‰ abundance in a given sample are displayed, whereas genera present in less than 5‰ are pooled in the corresponding phylum and referred to as “phylum_others.” Samples were characterized with regard to (i) quarter health status (Mq, has already developed mastitis; Hq, healthy (no mastitis at all); NDq, unknown status), and (ii) microbiological status at sampling time (I, infected; NI, not infected). The SCC of the quarter at the time of sampling is also included.

Table S5 **Differentially abundant taxonomic units between Cluster 1 (contains Mq quarters) and Cluster 2 (contains Hq quarters) as determined by the LEfSe pipeline**. Average and median abundances (expressed as percentages) as well as standard deviation are presented for each cluster. LEfSe results include LDA score and pval.
