# Supplementary figures and images for: Bovine Teat Microbiome Analysis Revealed Reduced Alpha Diversity and Significant Changes in Taxonomic Profiles in Quarters with a History of Mastitis
Source: Front Microbiol. 2016 Apr 8;7:480. doi: 10.3389/fmicb.2016.00480 (PMC4876361; doi:10.3389/fmicb.2016.00480)

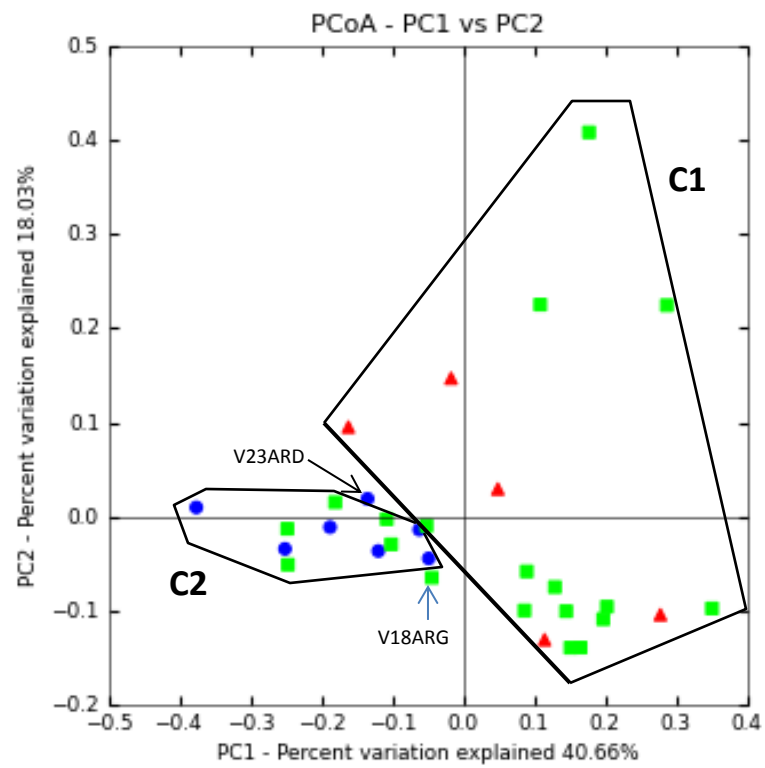

Supplement: Figure S1 — Principal Component Analysis based on weighted UniFrac distances. Samples are indicated by points and colored with regard to quarter health status: Hq, (blue); Mq, (red); NDq, (green). [file Presentation1.PDF]

## Slide 1
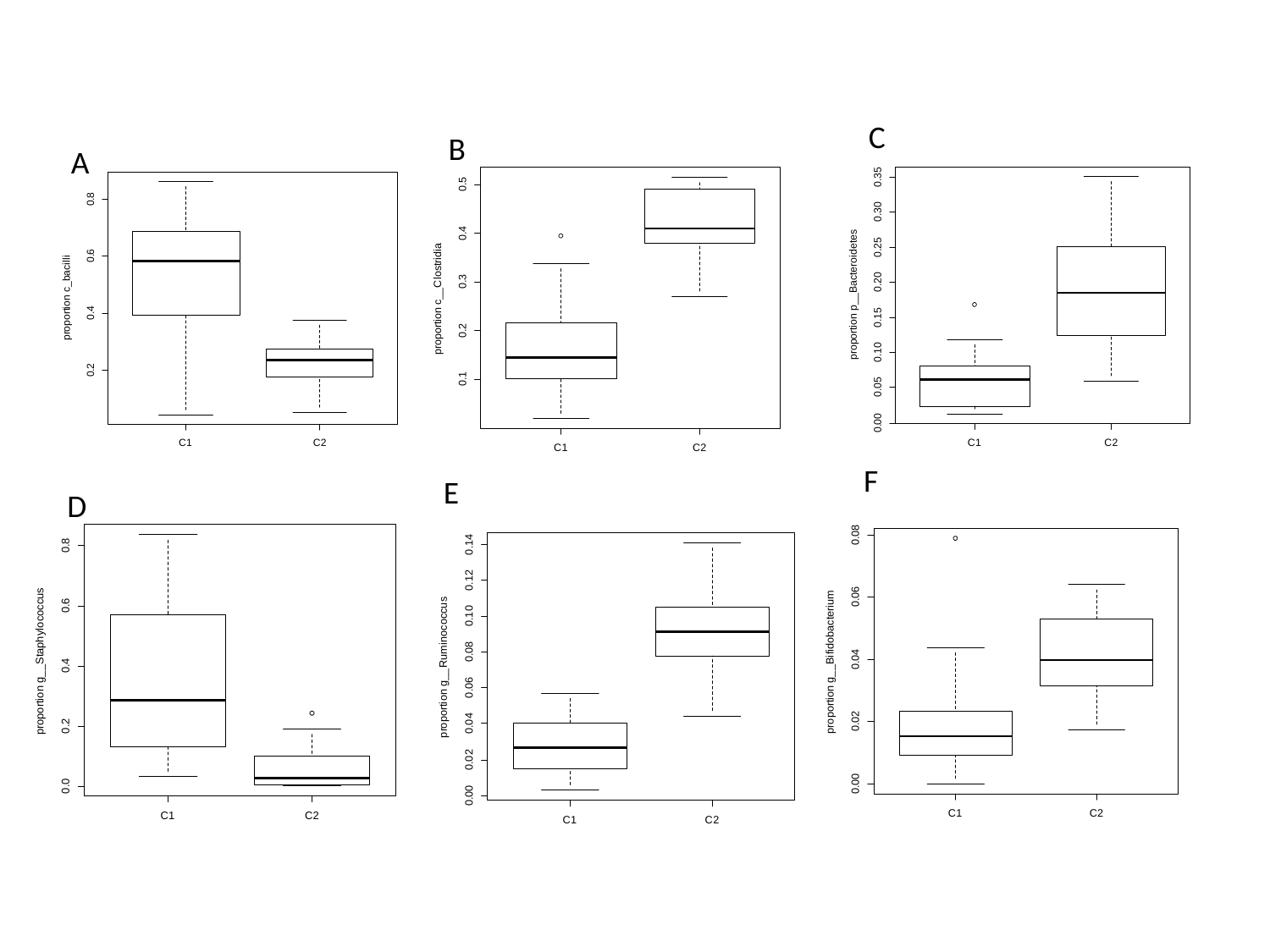

C
B
A
F
E
D

Supplement: Figure S2 — Selected boxplot of taxonomic units that are differentially abundant between Cluster 1 (contains quarters susceptible to mastitis) and Cluster 2 (contains healthy quarters) as determined by the LEfSe pipeline. The box represents the 75% (upper) and 25% (lower) quartiles and the black line inside the box represents the median. Abundance of the classes Bacilli (A) and Clostridia (B) the phylum Bacteroidetes (C) and the genera Staphylococcus (D) Ruminococcus (E) and Bifidobacterium (F). [file Presentation2.PPTX]
