# Supplementary material for: Bovine Teat Microbiome Analysis Revealed Reduced Alpha Diversity and Significant Changes in Taxonomic Profiles in Quarters with a History of Mastitis
Source: Front Microbiol. 2016 Apr 8;7:480. doi: 10.3389/fmicb.2016.00480 (PMC4876361; doi:10.3389/fmicb.2016.00480)
Supplement: Table S1 — Quarter status based on the history of the animal. [file Table1.DOCX]

**Table S1: Quarter status based on the history of the animal**

| **Quarter Status** | **Healthy (Hq)** | **Mastitis (Mq)** | **Not Determined (NDq)** |
| --- | --- | --- | --- |
| **Ongoing lactation** |  |  |  |
| SCC on the milk collected from the four quarters (animal level) (cells/ml) | <250 000 | <250 000 | <250 000 |
| SCC on the milk collected from the sampled quarter one week before sampling (cells/ml) | <100 000 | <100 000 | <100 000 |
| Clinical signs of mastitis on the sampled quarter | no | no | no |
| **Previous lactations** |  |  |  |
| SCC on the milk collected from the four quarters (animal level) (cells/ml) | <250 000 | At least one increase of SCC > 250 000 | At least one increase of SCC > 250 000 |
| Clinical signs of mastitis on the sampled quarter | no | yes | no |
